# Adulthood systemic inflammation accelerates the trajectory of age-related cognitive decline

**DOI:** 10.18632/aging.203588

**Published:** 2021-09-29

**Authors:** Jolie Barter, Ashok Kumar, Linda Bean, Marissa Ciesla, Thomas C. Foster

**Affiliations:** 1 Department of Neuroscience, McKnight Brain Institute, University of Florida, Gainesville, FL 32610, USA; 2 Genetics and Genomics Program, University of Florida, Gainesville, FL 32611, USA

**Keywords:** inflammation, LPS, hippocampus, synaptic function, longitudinal, NMDA receptor

## Abstract

In order to understand the long-term effects of systemic inflammation, it is important to distinguish inflammation-induced changes in baseline cognitive function from changes that interact with aging to influence the trajectory of cognitive decline. Lipopolysaccharide (LPS; 1 mg/kg) or vehicle was administered to young adult (6 months) male rats via intraperitoneal injections, once a week for 7 weeks. Longitudinal effects on cognitive decline were examined 6 and 12 months after the initial injections. Repeated LPS treatment, in adults, resulted in a long-term impairment in memory, examined in aged animals (age 18 months), but not in middle-age (age 12 months). At 12 months following injections, LPS treatment was associated with a decrease in N-methyl-D-aspartate receptor-mediated component of synaptic transmission and altered expression of genes linked to the synapse and to regulation of the response to inflammatory signals. The results of the current study suggest that the history of systemic inflammation is one component of environmental factors that contribute to the resilience or susceptibility to age-related brain changes and associated trajectory of cognitive decline.

## INTRODUCTION

Variability in the trajectory of age-related cognitive decline has stimulated research into the relationship between systemic inflammation and cognitive function. Polymorphisms in immune response-related genes [1–6] and elevated markers of systemic inflammation [7–11] have been associated with an increased rate of cognitive decline. In addition, severe acute systemic inflammation can result in long-lasting cognitive impairments, including increased susceptibility to neurodegeneration after resolution of the infection [12–14]. Concern about the history of inflammation has increased due to the COVID-19 pandemic and neurological features of the disease, which suggest possible long-term effects [15–19]. The results from studies in humans suggest that in order to understand the relationship between severe infections that arise in adulthood and cognitive impairment with advanced age, it is important to distinguish between changes in baseline verses a difference in the trajectory of cognitive decline [10, 11, 20–26].

Animal models indicate that inflammation induced by lipopolysaccharide (LPS) treatment, during a period of neurodevelopment or in young adulthood, may increase vulnerability to cognitive impairment with age or due to a subsequent occurrence of systemic inflammation through altered synaptic plasticity mechanisms [27–29]. One possibility is that early inflammation primes or trains the brain to increase responsiveness to a secondary immune stimulation by either a subsequent systemic inflammation or chronic low-level systemic inflammation associated with aging. In contrast, other studies indicate that LPS treatment in young adults result in immune tolerance in the brain due to long-lasting epigenetic changes, resulting in decreased expression of LPS-induced pro-inflammatory genes, cytokines, chemokines, and neurotrophic factors, and increased phagocytosis [30, 31]. The goals of this study was to longitudinally examine the effect of systemic inflammation, administered in young adult (6 months) male rats, on subsequent cognitive decline during aging, and related treatment effects on synaptic function and gene expression in the hippocampus.

## RESULTS

### Behavioral characterization at 12 months of age

**Figure 1:**
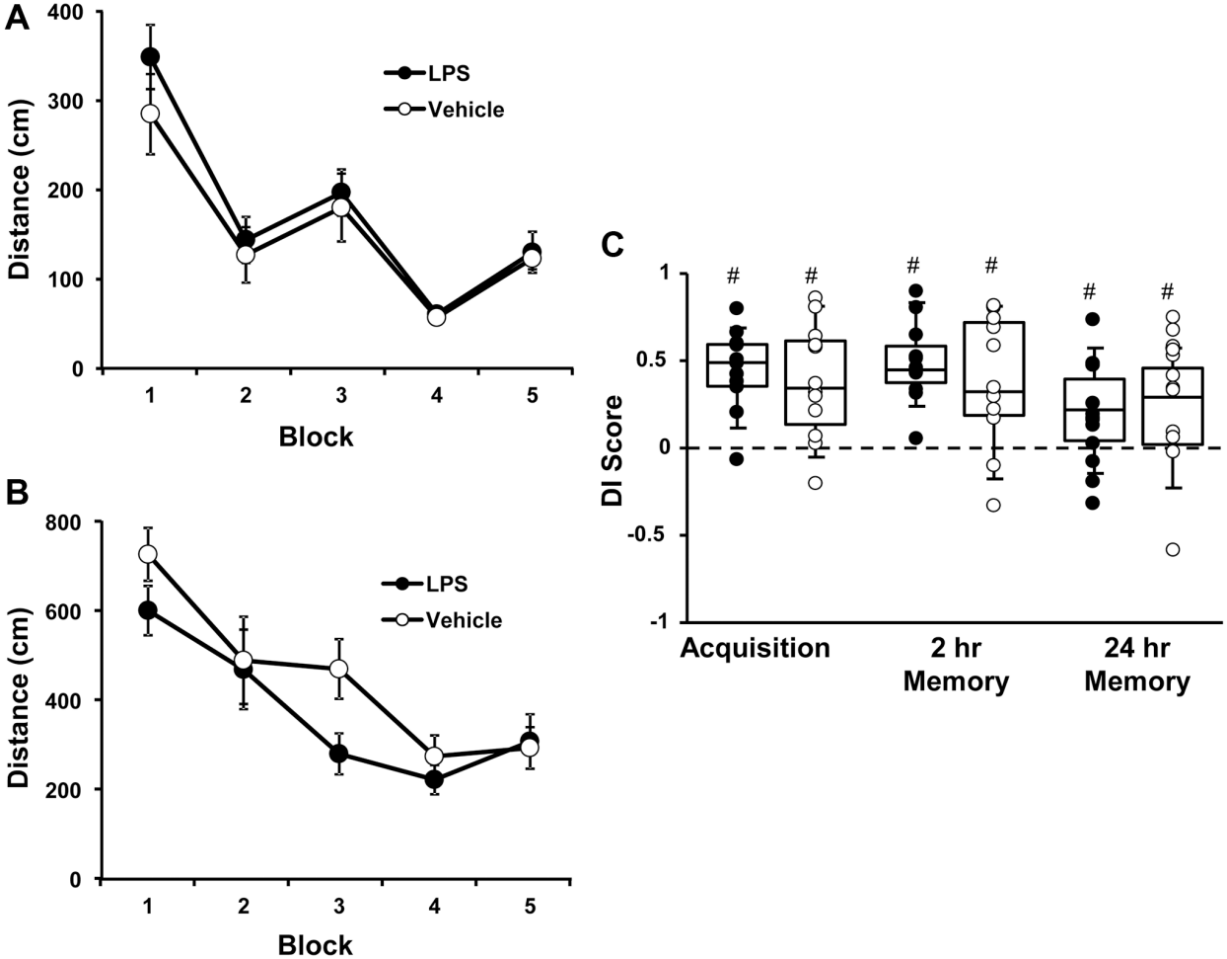
**Vehicle and LPS treated animals learn cue and spatial discrimination with no difference in learning or memory at 12 months, 6 months after treatment.** Symbols represent mean escape path length (± SEM) for vehicle (open circles) and LPS treated (filled circles) animals over the training blocks for (**A**) cue and (**B**) spatial discrimination. (**C**) Box and whisker plots and individual DI scores from the acquisition, 2 hr and 24 hr probe trials. Pound sign indicates significant difference from chance (*p* < 0.05).

At 6 months of age, rats were injected with vehicle (*n* = 12) or LPS (*n* = 12) once a week for 7 weeks. Cognitive function was first assessed at 12 months of age (6 months after the onset of injections). For the cue discrimination task, there was an effect of training [F(4, 80) = 18.76, *p* < 0.0001] in which animals swam less distance to find the platform over the blocks of training (Figure 1A), in the absence of a treatment effect. Similarly, for spatial discrimination training, there was an effect of training [F(4, 80) = 12.2, *p* < 0.0001] on the distance to find the platform (Figure 1B), in the absence of a treatment effect. A repeated measures ANOVA performed on the DI scores for the acquisition, 2-hr, and 24-hr memory probe trials indicated no effect of treatment and an effect of repeated testing [F(2, 44) = 3.848, *p* < 0.05]. *Post hoc* analysis indicated that the DI scores for the 24-hr probe trial were reduced relative to the acquisition and 2-hr retention probe trials (Figure 1C). Finally, one group *t*-tests, on the DI scores within each treatment group and probe trial, indicated performance was above chance for each group for each probe trial. The results indicate that, at middle-age, no group effects were observed for acquisition or retention of a spatial episodic memory, examined 6 months after the LPS injections.

### Behavioral characterization at 18 months of age

**Figure 2:**
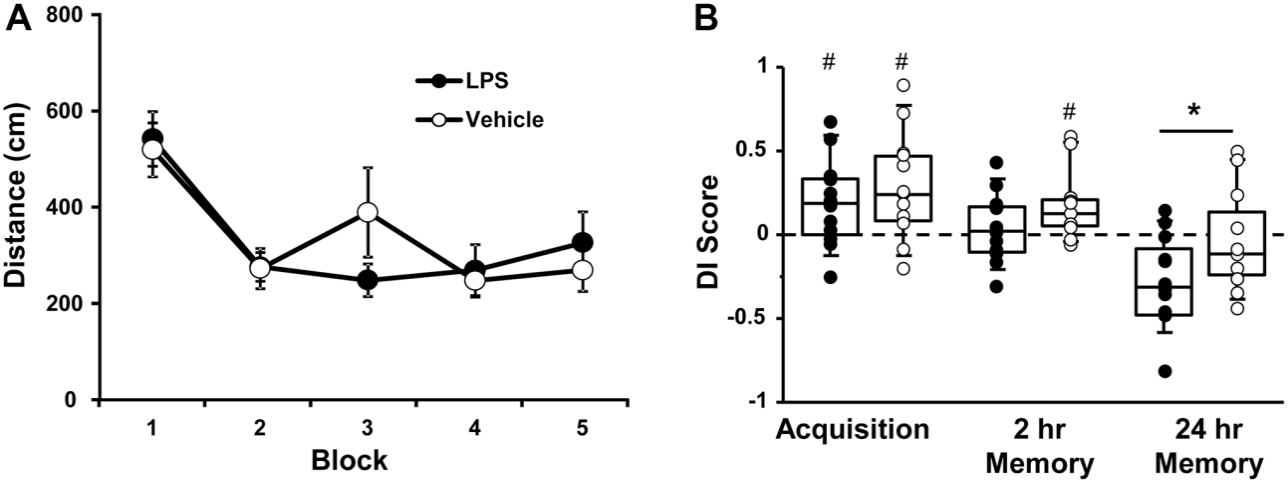
**Vehicle and LPS treated animals learn the spatial discrimination and differences in memory emerge at 18 months, 12 months after treatment.** Symbols represent mean escape path length (± SEM) for vehicle (open circles) and LPS treated (filled circles) animals over the training blocks for (**A**) spatial discrimination. (**B**) Box and whisker plots and individual DI scores from the acquisition, 2 hr and 24 hr probe trials. Pound sign indicates significant difference from chance (*p* < 0.05). Asterisk indicates significant treatment difference (*p* < 0.05).

At 18 months of age (12 months after the injections), the escape platform location was moved to a different location and rats were again assessed for acquisition and retention of a spatial episodic memory. A repeated measures ANOVA indicated an effect of training [F(4, 80) = 12.31, *p* < 0.0001], such that the distance to find the platform decreased over the training blocks, in the absence of a treatment effect (Figure 2A).

A repeated measures ANOVA on the DI scores indicated an effect of repeated testing [F(2, 44) = 17.65, *p* < 0.0001] and treatment [F(1, 22) = 5.0, *p* < 0.05]. *Post hoc* tests for the repeated testing indicated that the 24-hr DI scores were reduced compared to the acquisition and the 2-hr probe trials and the 2-hr probe trial was reduced relative to the acquisition probe. *Post hoc* tests for treatment effects on each probe trial indicated that LPS-treated animals performed poorly compared to vehicle treated for the 24-hr probe [F(1, 22] = 4.65, *p* < 0.05]. One-tailed *t*-test indicated that both groups performed above chance during the acquisition and only vehicle treated animals were above chance for the 2-hr probe trail (Figure 2B).

While LPS treated animals exhibited impaired retention relative to controls, it is clear that age also influenced retention over the 24-hr delay. The interaction of age (12 and 18 months) and treatment was examined for probe trial performance using repeated measures ANOVAs. For the acquisition probe trial, a repeated measures ANOVA indicated decreased performance with age [F(1, 22] = 4.5, *p* < 0.05] in the absence of a treatment effect. However, planned repeated measures ANOVAs within each group indicated an age effect for the LPS group [F(1, 11] = 6.23, *p* < 0.05], but not the vehicle group (*p* = 0.52). Similarly, for the 2-hr probe trial a repeated measures ANOVA indicated a significant effect of age [F(1, 22] = 17.46, *p* < 0.0005] in the absence of a treatment effect and planned repeated measures ANOVAs within each group indicated a significant effect of age for the LPS group [F(1, 11] = 20.19, *p* < 0.001], but not the vehicle group (*p* = 0.12). Finally, for the 24-hr retention probe trial, a significant effect of age [F(1, 22] = 16.86, *p* < 0.0005], was observed in the absence of a treatment effect and planned repeated measures ANOVAs within each group indicated a significant effect of age for the LPS group [F(1, 11] = 14.36, *p* < 0.005] and a trend for an age effect in the vehicle group (*p* = 0.07). Thus, while age-related memory deficits over 24-hrs were variable in vehicle treated animals, a robust decline in memory was observed over the course of aging for LPS treated animals.

### Hippocampal synaptic function

**Figure 3:**
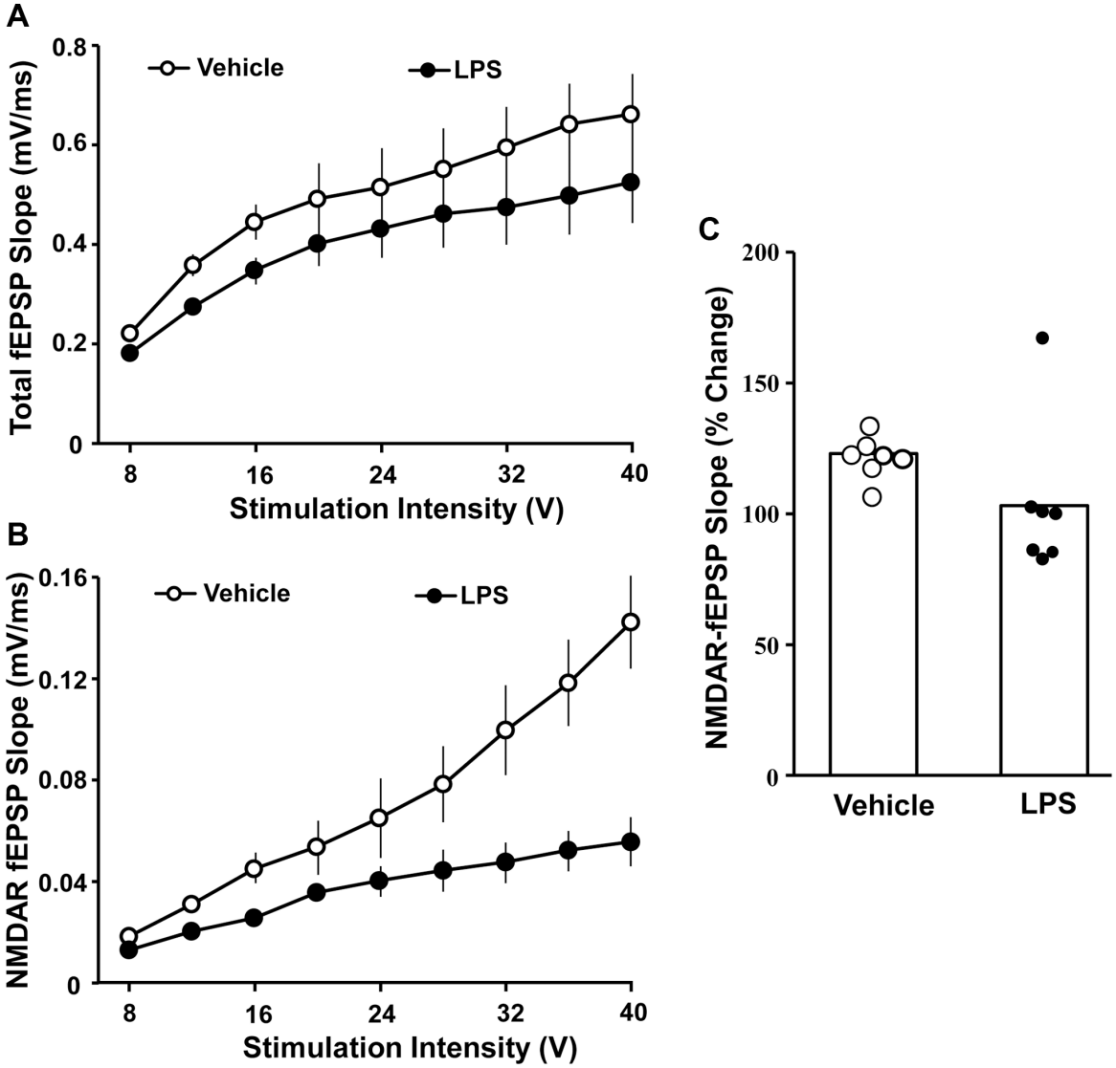
**Decreased NMDAR-mediated synaptic responses associated with prior LPS treatment.** Input-output curves for the mean slope (± SEM) of the total fEPSP (**A**) and NMDAR-fEPSP (**B**) evoked by increasing stimulation voltage (V). Data is presented for the vehicle (open circles) and LPS treatment (filled circles) recorded at 18 months, 12 months after the final LPS or vehicle injection. (**C**) Bars illustrating mean percentage change in NMDAR fEPSP slope induced by bath application of DTT in slices obtained from LPS (*n* = 7/4 slices/animal) or vehicle (*n* = 7/4 slices/animal)-treated animals. The distribution of individual responses is also depicted.

The effect of LPS exposure at 6 months of age on hippocampal synaptic transmission examined at 18 months of age was assessed in animals that were not behaviorally characterized. Hippocampal CA1-CA3 synaptic strength was examined by recording total-fEPSP and generating input-output curves and plotting the slope of total synaptic response across the different stimulation intensities for vehicle (*n* = 8/4 slices/animals) and LPS (*n* = 8/4 slices/animals) treated animals. A repeated-measures ANOVA performed across the stimulation intensities indicated an effect of stimulation intensity [F(7, 98) = 44.281, *p* < 0.0001]. Despite a general decrease in the synaptic response for LPS treated animals, no effect of treatment or interaction of treatment and stimulation intensity was observed for total synaptic response (Figure 3A). After assessing the total synaptic response, the NMDAR-mediated synaptic component was pharmacologically isolated. Again, we generated input-output curves for vehicle and LPS treated animals. For the NMDAR-mediated synaptic response, a repeated measures ANOVA indicated an effect of stimulation intensity [F(7, 98) = 42.774 *p* < 0.0001], treatment [F(1, 14) = 39.439, *p* < 0.0001], and an interaction of intensity X treatment [F(7, 98) = 26.346, *p* < 0.0001] due to a robust decrease in the NMDAR-mediated synaptic response in LPS treated animals compared to vehicle (Figure 3B).

The decline in the NMDAR-mediated component of synaptic transmission with age can result from a decline in receptors or a redox mediated hypofunction [32–34]. To determined possible differences in NMDAR function due to redox state, the reducing agent, DTT, was added to the bath after collection of baseline NMDAR-mediated synaptic responses and the response was collected for 1 hr in slices obtained from LPS (7/4 slices/animal) and vehicle (7/4 slices/animal) treated animals (Figure 3C). An ANOVA indicated no difference between groups; however, *t*-tests indicated that DTT application increased the synaptic response relative to baseline in slices from vehicle control animals [t (6) = 6.26, *p* < 0.001], while the post DTT response was not different from baseline in LPS treated animals (Figure 3C).

### RNA expression

**Figure 4:**
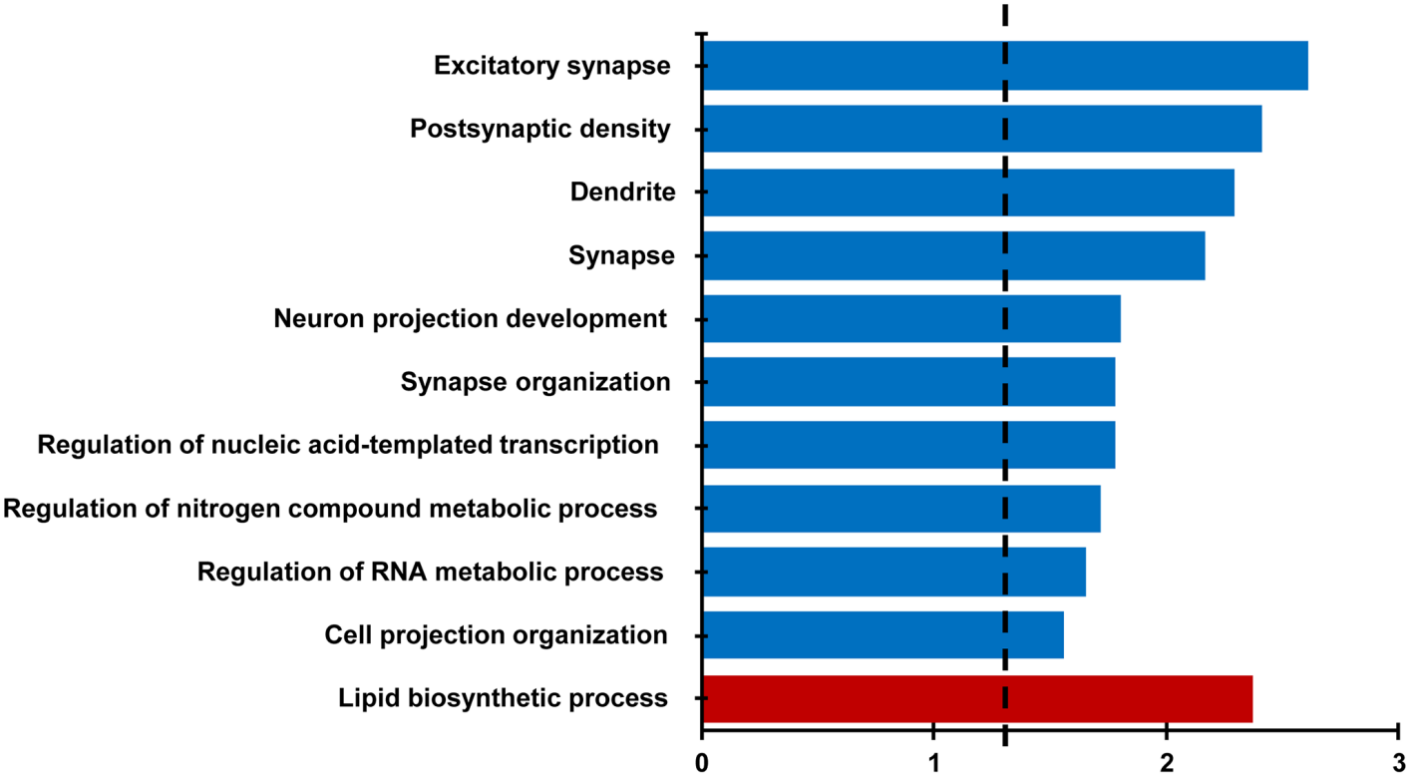
**Differential gene expression analysis evaluating the effect of treatment in the CA1 region.** Bars represent the –log (*p* value) for selected GO terms that were significant for down regulated (blue) and upregulated (red) genes. Dotted line is the –log (0.05).

For behaviorally characterized LPS (*n* = 8) and vehicle (*n* = 8) treated rats, RNA-sequencing was performed on the CA1 region of the hippocampus collected at 18 months of age. Differential expression filtering resulted in 157 genes that increased and 286 genes that decreased with treatment. For the genes that increased with LPS treatment, unsupervised functional annotation analysis indicated that these genes were enriched for lipid biosynthetic process (Figure 4, Table 1). Functional annotation clustering analysis for genes that were downregulated with LPS treatment were related to excitatory synapse, postsynaptic density, dendrite, synapse, neuron projection development, and synapse organization (Figure 4, Table 1). In order to examine possible candidate genes related to inflammation, we examined genes within the clusters response to lipopolysaccharide (GO:0032496), regulation of immune system process (GO:0002682), and inflammatory response (GO:0006954). The LPS treatment group exhibited decreased expression of genes that are normally upregulated by LPS, involved in the production of cytokines (*Adam8, Tnfrsf25, Comt, Bcr, Pml, Mavs, Nectin2, Dapk1*), or linked to regulation of the immune response (*Asb2, Cdkn1a, Cyp4f5, Dnaaf2, Mtor, Nthl1*), including transcription regulators (*Kdm6b, Tfe3, Tead4*). In contrast, upregulated genes were associated with phagocytosis and toxic effects of microglial activation (*Ripk1, Kcnn4, Snx4, Fcgr3a*). Interestingly, *Gng12*, a negative regulator of the LPS response was increased. Interestingly, no differences were observed for markers of activated microglia (*Aif1, Cd68*) and astrocytes (*Gfap*). The results confirm other studies that find memory impairment linked to aging is associated with a decrease in synaptic genes [35–37] and suggest that prior LPS treatment was associated with an altered expression of genes linked to inflammatory/immune response.

**Table 1:** Gene ontology categories and lists of differentially expressed genes evaluating the effect of treatment in the CA1 region.

**Category**	**Term**	**Count**	**Genes**	**Benjamini *p*-value**
GOTERM_BP	GO:0008610~lipid biosynthetic process	17	HMGCS1, NUS1, ST8SIA4, GPAT3, HSD17B7, INPP4B, INSIG1, IGF2, IDH1, MSMO1, PEX2, PDSS1, PTGDS, PTPMT1, SELENOI, SC5D, THRSP	0.004
GOTERM_CC	GO:0060076~excitatory synapse	15	BCR, MAGI2, BAIAP2, GRIK5, MINK1, BSN, PPP1R9B, SH2D5, SEMA4C, LRFN1, CAMK2B, NSMF, UNC13A, DISC1, ADD2	0.002
GOTERM_CC	GO:0014069~postsynaptic density	14	BCR, MAGI2, BAIAP2, GRIK5, MINK1, BSN, PPP1R9B, SH2D5, SEMA4C, LRFN1, CAMK2B, NSMF, DISC1, ADD2	0.004
GOTERM_CC	GO:0030425~dendrite	25	CRTC1, GRIK5, HCFC1, COMT, KCNJ2, ZMYND8, NUMA1, HTR1A, INPP5J, ANK3, AGO2, CAMK2B, HAP1, SLC8A2, MAGI2, BAIAP2, STRN4, MINK1, BSN, PPP1R9B, ADCY9, KHSRP, NSMF, RGS8, MTOR	0.005
GOTERM_CC	GO:0045202~synapse	28	CRTC1, GRIK5, COMT, KCNJ2, ZMYND8, AMPH, SH2D5, ANK3, LRFN1, CAMK2B, HAP1, DISC1, SLC8A2, MAGI2, BCR, BAIAP2, RIMBP2, STRN4, MINK1, BSN, PPP1R9B, DOK7, LRP6, SEMA4C, NSMF, DOC2B, UNC13A, ADD2	0.008
GOTERM_BP	GO:0031175~neuron projection development	29	CRTC1, ZMYND8, FOXO6, IGF1R, JADE2, FOLR1, UNC5A, INPP5J, ANK3, CAMSAP1, LRFN1, OBSL1, CAMK2B, MKL1, HAP1, DISC1, MAGI2, BAIAP2, LRRN2, SDK1, MINK1, NTNG2, ARID1B, PPP1R9B, SEMA4C, NSMF, MTOR, UNC13A, KIF26B	0.02
GOTERM_BP	GO:0050808~synapse organization	13	SEZ6L2, HTR1A, MAGI2, ANK3, MDGA1, DOK7, BSN, CAMK2B, SEZ6L, ZMYND8, UNC13A, DISC1, ADGRB2	0.02

### Western blot analysis

**Figure 5:**
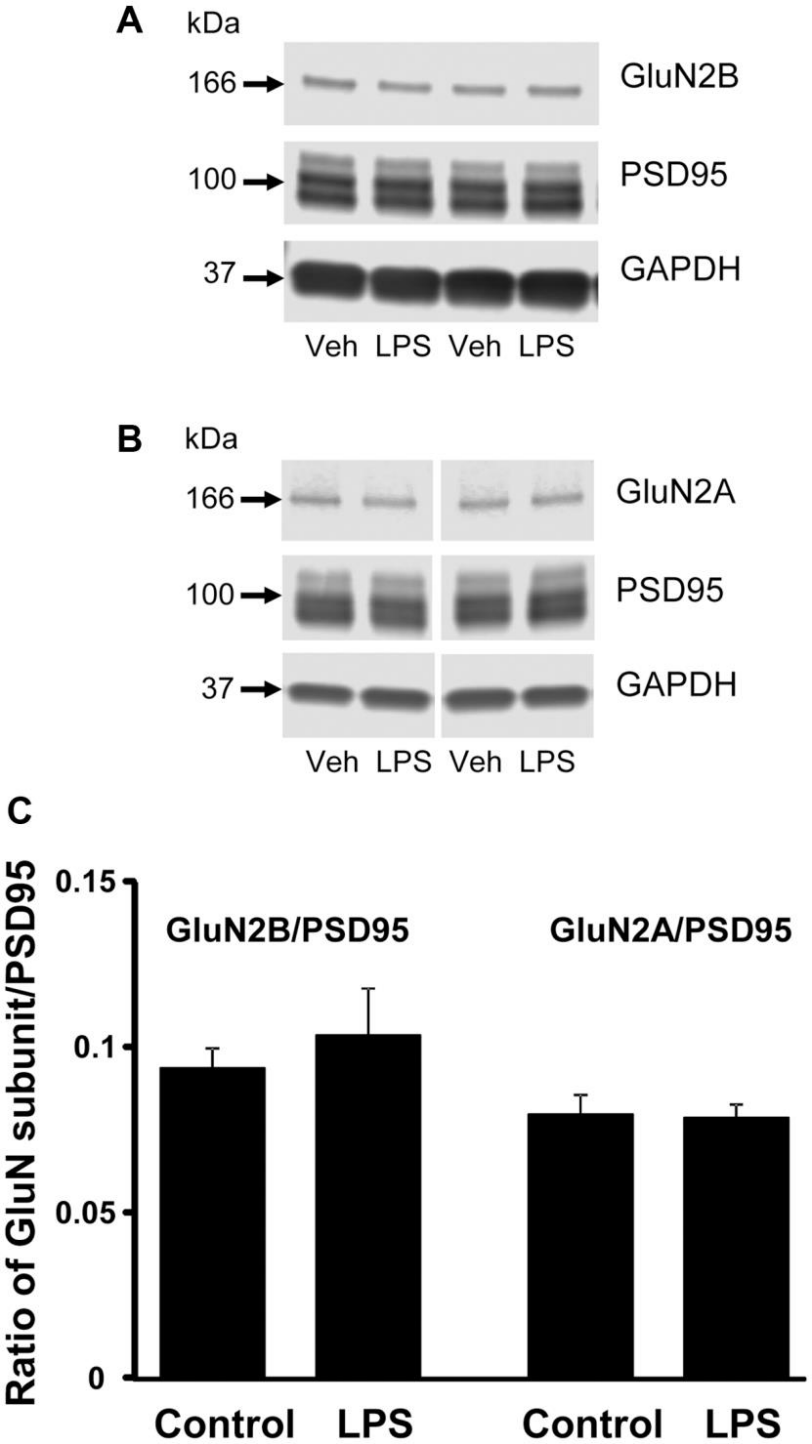
**Western blot analysis of NMDAR subunit expression in CA1 region of hippocampus.** The blots illustrate expression of (**A**) GluN2B, (**B**) GluN2A, and PSD95. (**C**) The bars represent the mean (± SEM) ratio of expression for vehicle (*n* = 4) and LPS treated (*n* = 4) animals.

CA1 tissue samples from LPS- (*n* = 4) and vehicle-treated (*n* = 4) behaviorally characterized animals were prepared for Western blot analysis. Membranes were immuno-stained for GluN2B, GluN2A, PSD95, and GAPDH (Figure 5). PSD95 signal intensities, normalized to GAPDH, were not significantly different between treatment groups (*p* = 0.66), which suggests no difference in synapse number. In addition, we found no significant group differences in the expression of either subunit of NMDAR, GluN2B and GluN2A normalized to GAPDH (*p* = 0.26 and *p* = 0.85 respectively, not shown), or for each subunit normalized to PSD95 expression (*p* = 0.52 and *p* = 0.89, respectively) (Figure 5).

## DISCUSSION

The current study indicates that inflammation in adulthood influences the trajectory of cognitive decline, from middle-age (12 months of age) to old age (18 months of age). At 18 months, 12 months after LPS injections, both groups exhibited a similar level of learning observed as a decrease in the distance to find the platform over the course of training. Furthermore, no difference was observed for the acquisition probe trial and both groups performed above chance indicating similar acquisition of a spatial search strategy. For retention probe trials, only the vehicle control group performed above chance for the 2-hr retention period. Also, the DI score for the 24-hr retention probe trial was decreased in LPS animals relative to vehicle controls. Furthermore, repeated measures analysis indicates that the age-related decline in cognition from 12 to 18 months was mainly due to LPS treated animals. The current study adds to the previous literature by demonstrating that inflammation initiated in adults, interacts with aging, contributing to the trajectory of cognitive decline, such that LPS treated animals exhibit greater susceptibility to memory deficits from middle-age to old age.

Age-related memory impairment is associated with a decline in NMDAR function [38] and impaired memory following LPS treatment in adults is also associated with decreased NMDAR function [35]. The current study provides the first evidence that systemic inflammation can have long-term effects on NMDAR synaptic function. Previous research in neonates suggest a shift in NMDAR subunit expression and the direction is dependent on when systemic inflammation occurred during development/maturation. Systemic inflammation initiated in neonates increased NMDAR subunit expression, particularly in the dentate gyrus, while inflammation initiated in adults resulted in a decrease in hippocampal NMDAR subunits [29, 39]. We did not observe a change in expression of GluN2B or GluN2A protein. However, we cannot rule out that the decrease in the NMDAR synaptic response was due to a decrease in the GluN1 subunit or altered plasticity processes that regulate the localization of NMDARs to the synapse.

Work from several labs indicates that the age-related NMDAR hypofunction is due to redox regulation of NMDARs [32, 40–44] involving translocation of NMDARs into the synapse [34]. We observed that addition of the reducing agent, DTT, increased the NMDAR synaptic response in aged, vehicle-treated animals. The inability to increase the NMDAR response of LPS treated animals under reducing conditions suggests alterations in the molecular machinery for NMDAR plasticity relative to that observed for normal aging [35].

For genes that passed our statistical filter, unsupervised analysis of gene enrichment indicated that LPS-treatment was associated with decreased expression of genes linked to synaptic function (excitatory synapse, postsynaptic density, dendrite, synapse, and synapse organization). The decrease in synaptic gene expression is consistent with work indicating that age-related cognitive decline is associated with decreased expression of synaptic genes [36, 37, 45]. However, it is unclear if the gene changes contribute to the decline in NMDAR synaptic transmission. It is also possible that altered expression represents an attempt to compensate for the decrease in NMDAR function/plasticity [38]. In previous studies examining the transcriptional profile of young adults treated with LPS, an increase in expression of synaptic genes was observed, suggesting a recovery/resilience process for young animals in response to the decline in synaptic transmission [35, 46]. Several of the recovery/resilience synaptic genes (*Sema4c, Sez6*) were down regulated in the current aged-LPS treated group and other down regulated synaptic genes (*Camk2b, Crtc1, Mink1*) were reported to decrease in aged cognitively impaired animals [35]. Taken together, the results suggest that the history of neuroinflammation contributes to the loss of synaptic function and decreased synaptic gene transcription during aging, possibly augmenting a loss of resilience in the face of aging stressors.

LPS treatments can have long-term effects on the brain’s response to inflammatory signals, possibly through epigenetic regulation of transcription. Acute peripheral inflammation results in immune priming or training, increasing the brains inflammatory response to a subsequent bout of systemic inflammation. In contrast, repeated LPS injections induce immune tolerance of microglia, which can be detected weeks or months following LPS injections [30, 31]. In the current study, several of the transcriptional changes associated with repeated LPS injections are consistent with an immune tolerance. Relative to vehicle controls, animals previously treated with LPS exhibited decreased expression of genes that are normally upregulated by LPS or the production of cytokines including those involved in signaling in response to LPS, the Tumor necrosis factor (TNF) family receptor, *Tnfrsf25*, and immunoreceptor (*Bcr*). Similarly, down regulation was observed for genes that promote cytokine production (*Pml*, and *Dapk1, Mavs*) [47, 48], regulate the cytokine response (*Comt*) [49–51], and mediate signaling downstream of cytokine activation (*Cdkn1a, Adam8, Ripk1*) [52–54]. Moreover, upregulated genes included *Gng12*, a negative regulator of the LPS response [55]. Immune tolerance of the brain might be beneficial in the face of chronic or repeated inflammation-inducing conditions. However, cytokines have a biphasic function on cognition, wherein low-levels improve and high-levels impair memory function [56–59]. Thus, a shift in mechanisms for cytokine signaling, either increasing or decreasing signaling, could impair memory.

Other upregulated genes suggest an increase in phagocytosis, potassium currents, and release of nitric oxide, which are observed following an inflammatory challenge in immune tolerant microglia [31]. In particular, there was an increase in expression of the potassium channel *Kcnn4*, which regulates the release of nitric oxide [60–64]. Increased expression was also observed for the cell-surface phagocytosis receptor, *Fcgr3a*, and genes linked to autophagy (*Atg12, Snx4*), antigen presentation pathway (*RT1-CE6*), Itm2a, involved in immunoglobulin production, and *Oas1g*, an immune response protein against viral infection. Finally, decreased expression was observed for epigenetic and transcription factor regulators, including those involved in inflammation (*Kdm6b, Tfe3, Tead4*) and DNA base excision repair (*Nthl1*), suggesting that inflammation in adulthood may have triggered epigenetic mechanism resulting in long-term changes in responsiveness to cytokines and inflammatory signals. Finally, it is possible that behavioral and brain differences were due to LPS induced cell senescence and the release of metabolic waste and toxic factors from other tissues.

Finally, it is possible that behavioral and brain differences were due to the history of systemic inflammation interacting with the age-related increase in chronic low level systemic inflammation, sometimes referred to as “inflammaging”. A bout of severe systemic inflammation can induce senescence of peripheral cells, including cells of the immune system [65–69]. In turn, senescent cells exhibit a senescence associated secretory phenotype (SASP), releasing toxic factors: pro-inflammatory cytokines, chemokines, extracellular matrix proteases, and microRNA in extracellular vesicles, that contribute to age-related diseases. Furthermore, senescent cells exhibit hyper-activation in response to a variety of inflammatory mediators, increasing the release of pro-inflammatory cytokines and chemokines [70]. If the history of infection alters the number or responsiveness of peripheral senescent cells, this could influence the level of inflammaging, which has been linked to cognitive impairment in humans [7–11] and animal models [35, 71, 72]. Future studies should consider examining the relationship between the history of infection on measures of senescent cells or markers of systemic inflammation, as a measure of biological aging that influences the trajectory of cognitive decline.

In summary, repeated LPS treatment in adults was associated with long-term effects on the trajectory of age-related cognitive decline, NMDAR function, and expression of genes linked to the synapse and regulation of the response to inflammatory signals. The results of the current study suggest that the history of systemic inflammation is one component of environmental factors that contribute to the resilience or susceptibility to age-related brain changes and associated trajectory of cognitive decline.

## MATERIALS AND METHODS

### Animals

Young (*n* = 32, 6 months) male Fischer 344 X Brown Norway hybrid rats were obtained from the National Institute on Aging colony through the University of Florida Animal Care and Service facility. Animals were housed in pairs on a 12:12 light/dark cycle (lights on at 6 PM). All procedures involving animals were approved by Institutional Animal Care and Use Committee at the University of Florida and were in agreement with guidelines recognized by the U.S. Public Health Service Policy on Humane Care and Use of Laboratory Animals.

### Experimental paradigm

**Figure 6:**
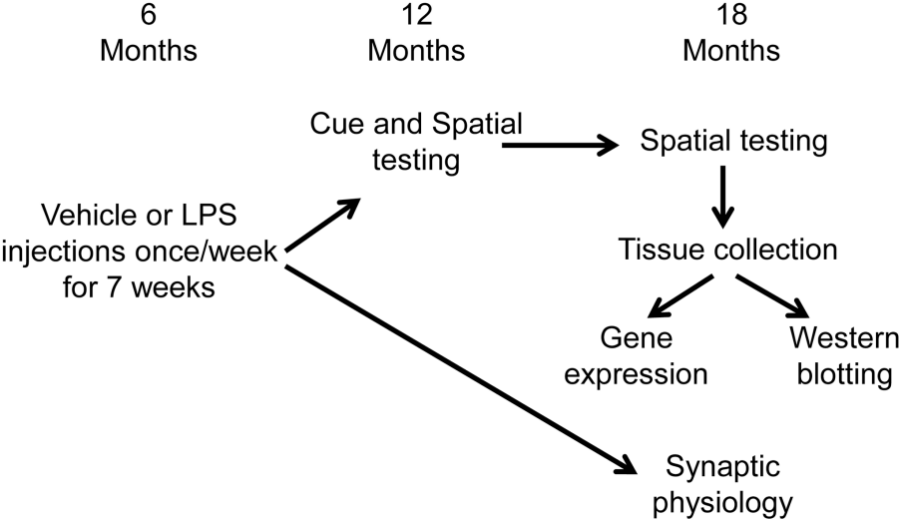
**Schematic representing the experimental paradigm for the longitudinal effect of systemic inflammation on cognition.** Young (6 months) male Fischer 344 X Brown Norway hybrid rats were either injected with vehicle (*n* = 16) or LPS (*n* = 16) once a week for 7 weeks. A subset of animals (vehicle *n* = 12; LPS *n* = 12) were cognitively assessed on the spatial discrimination water maze task at 12 and 18 months of age. Hippocampal tissue from behaviorally characterized rats was collected one week after completion of behavioral testing, at 18 months of age, and RNA sequencing was performed on the CA1 region of the hippocampus. The other group of animals (vehicle *n* = 4; LPS *n* = 4) were not behaviorally characterized and were used for electrophysiological experiments at 18 months of age (12 months after the injections).

Figure 6 illustrates the experimental timeline used to assess the longitudinal effect of LPS on spatial learning and memory. Following arrival, animals were acclimated to the new environment for one week, then administered intraperitoneal injections of LPS (1 mg/kg; *n* = 16) or vehicle (*n* = 16) once a week for 7 weeks. Our previous work indicates that this procedure induces a cognitive impairment, reduces the hippocampal CA3-CA1 synaptic response, and alters transcription examined days after the last injection [35]. A subset of these animals (LPS *n* = 12, vehicle *n* = 12) were cognitively assessed on the cue and spatial versions of the water maze tasks at 12 months of age (6 months after the onset of injections). The cue version of the water maze task was performed only at 12 months, since procedural memory for how to perform the swim task (i.e., how to swim and that the pool wall is not an escape route) is retained across the lifespan [73–75]. These same animals were again assessed on the spatial version of the water maze task at 18 months of age (12 months after the injections). Another subset of animals (LPS *n* = 4, vehicle *n* = 4), were not behaviorally characterized and were used for electrophysiological experiments at 18 months of age (12 months after the injections).

### Electrophysiology

Methods for collection of hippocampal slices and recordings have been published previously [34, 40, 76]. Briefly, rats were anesthetized with isoflurane (Halocarbon Laboratories, River Edge, NJ, USA) and swiftly decapitated. The brains were rapidly removed and the hippocampi were dissected. Hippocampal slices (~400 μm) were cut parallel to the Alvear fibers using a tissue chopper. The slices were incubated in a holding chamber (room temperature) containing standard artificial cerebrospinal fluid (aCSF) (in mM): NaCl 124, KCl 2, KH_2_PO_4_ 1.25, MgSO_4_ 2, CaCl_2_ 2, NaHCO_3_ 26, and glucose 10. Thirty to sixty minutes before recording, 2–3 slices were transferred to a standard interface recording chamber (Harvard Apparatus, Boston, MA, USA). The chamber was continuously perfused with standard oxygenated (95% O_2_, 5% CO_2_) aCSF at a flow rate of 2 ml/min. The pH and temperature were maintained at 7.4 and 30 ± 0.5°C, respectively. Humidified air (95% O_2_, 5% CO_2_) was continuously blown over the slices.

The total extracellular synaptic field potential (total-fEPSP) from CA3-CA1 hippocampal synaptic contacts were recorded with a glass micropipette (4–6 MΩ) filled with aCSF. Concentric bipolar stimulating electrodes (outer pole: stainless steel, 200 μm diameter; inner pole: platinum/iridium, 25 μm diameter, Fredrick Haer and Co, Bowdoinham, ME, USA) were positioned on approximately 1 mm of a recording electrode localized to the middle of stratum radiatum to stimulate CA3 inputs onto CA1. Using an SD9 stimulator (Grass Instruments, Braintree MA, USA), field potentials were induced by single diphasic stimulus pulses (100 μs). The signals were amplified, filtered between 1 Hz and 1 kHz, and stored on computer disk for off-line analysis (Data Wave Technologies, Longmont, CO, USA). The N-methyl-D-aspartate receptor (NMDAR)-mediated component of synaptic transmission (NMDAR-fEPSP) was obtained by incubating the slices in aCSF that contained low magnesium (Mg2^+^) (0.5 mM), 6,7-dinitroquinoxaline-2,3-dione (DNQX, 30 μM), and picrotoxin (PTX, 10 μM) [40, 71, 77]. Input-output curves for the total and NMDAR fEPSP (mV/ms) were constructed for increasing stimulation intensities.

The reducing effect of dithiothreitol (DTT) (0.5 mM) on NMDAR-fEPSP was performed by setting a baseline response at 50% of the maximum and the responses were collected for at least 10 min before and 60 min after drug application.

### Spatial water maze

For behaviorally characterized animals, one week before behavioral testing, animals were handled to allow for acclimation to the new environment. The water maze tasks were tracked on the Ethovision computer software (Noldus Information Technology, Leesburg, VA, USA). The water was dyed white (Rich Art-Tempera Paint), which allowed for the animals performance to be tracked. The pool was surrounded by a black curtain.

#### Cue discrimination testing

The animals were first habituated to the pool by freely swimming for 30 seconds followed by a gentle guidance to the platform. After habituation, the cue version of the water maze task was performed, which assesses sensory-motor performance and permits animals to learn the procedural aspects of the task, minimizing thigmotaxis [78]. During the cue task, the platform was topped with a white flag and was 1 cm above the water level. For cue training, animals completed five blocks of three trials (15 total trials) massed into a single day. For each trial, the animal was placed into the water at a randomly assigned release point. The animal was given 60 seconds to find the randomly assigned platform. If the animal did not locate the platform within 60 seconds, the animal was gently guided to the platform.

#### Spatial discrimination testing

Three days after cue discrimination testing, animals were tested on the one-day version of the spatial water maze, in accordance with previously described methods [35]. Briefly, objects were attached to the black curtain surrounding the pool to act as extra-maze cues. The platform location remained the same across all trials and was submerged 1.5 cm under the water level. For each trial, the animal was placed into the water from a randomly assigned release point and given 60 seconds to find the platform. If the animal could not locate the platform, the animal was gently guided to the platform. Spatial training consisted of 4 training blocks of three trials (12 total trials). An acquisition probe trial was performed between blocks 4 and 5 to assess if animals had learned the platform location. For a probe trial, the platform was removed and the animal was released from the quadrant opposite of the platform (goal) location and allowed to swim for 60 seconds. Following the acquisition probe trial, another training block was performed (block 5). In addition, memory probe trials were delivered 2 and 24 hours after the acquisition probe trial. When animals performed the spatial discrimination task for the second time, at 18 months, the escape platform was moved to a new quadrant. To analyze performance on the probe trials, a discrimination index (DI) score was calculated. A DI score measures the time spent in the goal quadrant (contains platform) compared to the opposite quadrant [(Goal Quadrant-Opposite Quadrant)/(Goal Quadrant + Opposite Quadrant)].

### Tissue collection

One week after completion of behavioral testing rats were removed from the home cage, anesthetized with isoflurane (Halocarbon Laboratories, River Edge, NJ, USA) and swiftly decapitated. The brains were rapidly removed and the subregions of the hippocampi were dissected. Samples were flash-frozen in liquid nitrogen.

### RNA, library preparation, and sequencing

RNA-sequencing was performed on the hippocampal subregion CA1 from behaviorally characterized LPS (*n* = 8) and vehicle (*n* = 8) animals using the Ion Proton system. RNA was isolated using the RNeasy Lipid Tissue Mini kit (Qiagen, Hilden, Germany, catalog number 74804) with DNase digestion with RNase-Free DNase Set (Qiagen, catalog number 79254). A NanoDrop 2000 spectrophotometer was used to measure RNA concentration and a High Sensitivity (HS) RNA Screen Tape in an Agilent 2200 Tapestation system was used to quantify the RNA integrity number (RIN). As a control for library preparation, External RNA Controls Consortium (ERCC) spike-in controls (Thermo Fisher Scientific, catalog number 4456740) were added to samples. To select for poly-(A) mRNA, the Dynabead mRNA DIRECT Micro kit (Thermo Fisher Scientific, catalog number 61021) was used. Ion Total RNA-seq Kit v2 (Thermo Fisher Scientific, catalog number 4475936) was used to prepare libraries. For multiplex sequencing, Ion Xpress barcodes (Thermo Fisher Scientific, catalog number 4475485) were added. The concentration and size distribution of the libraries were assessed using the Qubit double-stranded DNA HS Assay (Thermo Fisher Scientific, catalog number 32851) and HS D1000 Screen Tape in a Tapestation system. An Ion Chef system was used for template preparation and sequenced on an Ion Proton system. Using the ERCC analysis plugin on the Torrent Server, the ERCC analysis was performed and found that samples contained at least 40 transcripts with an R^2^ of above 0.9. Each sample contained about 30 million reads of 145 base-pair length.

### Bioinformatics and statistical analyses

Data analysis was performed using the Partek Flow server. FASTQ files were trimmed based on quality score and then aligned to the rat genome (rn6) using STAR. In Partek, gene-level counts were generated and annotated. DESeq2 was used to normalize genes. Genes for cluster analysis were filtered in two steps. First, genes with an average of less than 5 reads were removed [45, 79–81]. Second, statistical filter was used to generate a list of genes for cluster analysis. A *p* < 0.05 was used as a statistical filter to select differentially expressed genes (DEGs) between LPS treated animals compared to control. To assess the relationship of gene expression and cognitive performance, Spearman correlations were calculated between normalized counts of gene expression and the DI score measured during the 2-hr probe task. The criterion for a significant correlation was set at *p* < 0.05, consistent with our previous work [80].

Confidence in the significance of individual genes is low due to false positives associated with multiple comparisons across all genes. Gene enrichment analysis was performed under the assumption that changes in biological processes with treatment or cognition would result in a shift in the expression of clusters of genes related to the biological process [36]. Therefore, filtered genes were separated based on the direction of change and submitted to the National Institute of Health (NIH) Database for Annotation, Visualization, and Integrated Discovery (DAVID) for gene enrichment and functional annotation clustering analysis [36, 79, 80, 82, 83]. For unsupervised analysis, we report clusters for Gene Ontology (GO) terms for Cellular Components (CC), Biological Process (BP), and Kyoto Encyclopedia of Genes and Genomes (KEGG) pathways that exhibited a Benjamini False Discovery Rate (FDR) *p* < 0.05. In addition, we conducted directed analysis of several GO terms based on previous research examining age-related cognitive impairment and the hypothesis that prior LPS treatment would influence neuroinflammation responsiveness. We focused specifically on the synapse (GO:0045202), response to lipopolysaccharide (GO:0032496), regulation of immune system process (GO:0002682), and inflammatory response (GO:0006954).

### Western blot analysis

The CA1 region of the dorsal hippocampus from behaviorally characterized LPS (*n* = 4) and vehicle (*n* = 4) animals were isolated, flash frozen in liquid nitrogen, and stored at −80°C. Tissue samples were sonicated and lysed in radio-immunoprecipitation assay (RIPA) buffer (Thermo Fisher Scientific cat#89900) supplemented with phosphatase inhibitors, protease inhibitors and EDTA (Thermo Fisher Scientific, Waltham, MA, USA) and centrifuged at 20,000 × *g* for 10 minutes at 4°C. Protein concentrations were measured using Pierce BCA protein assay (Thermo Fisher Scientific cat#23227). Lysates were combined with 2×-Laemmli sample buffer (Bio-Rad cat#1610737) containing 2-mercaptoethanol (Sigma cat#M3148) and boiled at 97–98°C for 5 minutes prior to electrophoresis. Samples (10 μg per well) and Chameleon Duo protein ladder (Li-Cor cat#928-60000) were separated on 4%–15% Criterion TGX stain-free gels (Bio-Rad cat#5678085) in running buffer (Tris/Glycine/SDS, Bio-Rad cat#1610732) at 80V/20 min, 100V/45 min, and 120V/30 min. Prior to transfer, filter papers, membranes, and gels were equilibrated in ice-cold transfer buffer (10× Tris/Glycine, Bio-Rad cat #1610771) containing 20% methanol. Proteins were transferred to low fluorescent polyvinylidene fluoride membranes (LF-PVDF, Bio-Rad cat#1620262) using a Criterion Blotter with plate electrodes (Bio-Rad, Hercules, CA, USA) at 100V/30 minutes. The immunoblots were washed in TBS and blocked with Intercept (TBS) Blocking Buffer (Li-Cor P/N 927-60001) for 1 hour at room temperature followed by overnight incubation at 4°C with primary antibodies diluted with Intercept T20 (TBS) Antibody Diluent (Li-Cor P/N 927-65001). Antibodies used were anti-GluN2B, mouse monoclonal (Millipore/Sigma, 05-920) 1:1000; anti-GluN2A, rabbit polyclonal (Invitrogen, A-6473) 1:500; anti-PSD95, mouse monoclonal (Thermo Fisher Scientific, MA1-045) 1:1000 and anti-GAPDH, mouse monoclonal (Encor Biotechnology, MCA1D4) 1:10,000. Membranes were washed 3 times in TBST prior to a one hour room temperature incubation with IRDye 800CW and 600LT secondary antibodies (1:20,000) diluted with Intercept T20 (TBS) Antibody Diluent. Membranes were washed with TBST 3 times for 10 minutes each, and then rinsed 3 times with TBS before scanning on Li-Cor Odyssey CLx Imaging System. The data were analyzed with Image Studio Lite Ver 5.2. Target protein signals were normalized to the expression of the house-keeping protein, GAPDH. Technical replicates (×2) for signal intensity were averaged across blots.

### Statistical analysis for electrophysiology, behavior, and western blot data

ANOVAs were employed to examine input/output curves and treatment effects for synaptic responses. Similarly, repeated measures ANOVAs were used to examine treatment differences across training blocks for cue or spatial discrimination testing and across probe trial DI scores within each test period, 12 and 18 months. In addition, repeated measures ANOVAs were used to examine aging and LPS treatment on behavioral measures across the 12 and 18 month time points. Significant differences were localized using Fischer’s PLSD *post hoc* comparisons (*p* < 0.05). In addition, due to the prediction that LPS-treatment would impair cognition, *post hoc* ANOVAs within each treatment group were employed to determine if significant effects were driven by LPS or vehicle control animals. One-tailed one-group *t*-tests (*p* < 0.05) were performed to determine if the DI scores were above that expected by chance (i.e., DI score = 0) and for DTT studies to determine if the synaptic response increased above baseline.
